# A Rare Presentation of Acute Renal Failure Secondary to Rhabdomyolysis in a Patient Due to Atorvastatin Requiring Short-Term Renal Replacement Therapy

**DOI:** 10.7759/cureus.23511

**Published:** 2022-03-26

**Authors:** Zahid Khan, Mildred Ibekwe, Mohammed Abumedian, Yousif Yousif, Gideon Mlawa

**Affiliations:** 1 Cardiology, Royal Free Hospital, London, GBR; 2 Internal Medicine, Barking, Havering and Redbridge University Hospitals National Health Service (NHS) Trust, London, GBR; 3 Geriatrics, Barking, Havering and Redbridge University Hospitals National Health Service (NHS) Trust, London, GBR; 4 Internal Medicine and Diabetes and Endocrinology, Barking, Havering and Redbridge University Hospitals National Health Service (NHS) Trust, London, GBR

**Keywords:** renal calculi, ganglioneuroma – retroperitoneal – imaging – pyelonephritis, non-anion gap metabolic acidosis, creatinine kinase. acute kidney injury, statin induced rhabdomylosis, rhabdomyolysis, drug-related side effects and adverse reactions, non-oliguric renal failure, acute renal failure and hemodialysis in icu

## Abstract

Renal failure secondary to rhabdomyolysis due to statins is quite rare. We present a case of a 57-year-old patient who developed acute renal failure due to rhabdomyolysis secondary to atorvastatin. Interestingly, this patient had a similar presentation 27 years ago requiring dialysis only once resulting in complete resolution of symptoms. He presented to the hospital generally feeling unwell and then developed generalized body ache. He had an extremely elevated creatinine kinase level of 116,000 and it went up to 145,000. His urine dip was negative for nitrites and was positive for blood and protein. He was commenced on intravenous fluids. He also had a computerized tomographic scan of the kidneys, ureters, and bladder, which showed some fat stranding around both kidneys likely inflammatory in origin. His creatinine level continue to rise despite intravenous fluids and was acidotic on blood gases. He also tested positive for COVID-19 on day 7 of admission and eventually needed dialysis. His renal functions improved to baseline post dialysis and kidney functions returned to normal. His autoimmune screen was negative and his renal functions remained normal on a follow-up visit.

## Introduction

Statins belong to the group hydroxy-methylglutaryl coenzyme A (HMG-CoA) reductase inhibitors and have become the most widely prescribed drug worldwide since its introduction in 1987 with approximately, 25 million patients were on statin medications in 2008 [1,2]. Statins are effective lipid-lowering drugs that result in reduced cardiovascular morbidity and mortality, and although they are generally safe but can result in serious adverse effects rarely. As the indications for statins use have increased, so has the prescription and more patients are on statins today than ever before, which has resulted in a higher risk of side effects and there is not a single reliable predictive tool that can provide an accurate estimation of risk of developing these serious adverse effects [2].

Rhabdomyolysis is a very serious but rare complication of lipid-lowering therapy particularly statins, and the risk is increased by combination therapy of statin and fibrate likely due to pharmacodynamic interactions. The risk is also increased by advancing age and comorbidities such as diabetes, hypothyroidism, renal impairment, and polypharmacy due to drug interactions again [3]. The main feature of rhabdomyolysis is the release of myoglobin into the bloodstream by muscle necrosis, which can manifest from an asymptomatic elevation of serum muscle enzymes to serious complications such as renal failure requiring renal replacement therapy (RRT) [1].

Statins in general are quite well-tolerated and have mostly minor side effects however the two serious side effects include hepatotoxicity and injury to skeletal muscles that can range from myalgia to myopathy [4]. In the case of myopathy, the serum creatinine kinase (CK) level can increase more than 10-fold at least and occurs in about 0.5% to 1% of patients and the severity is mostly dose-dependent [4]. About 0.04% to 0.2% of patients from this percentage of patients develop rhabdomyolysis and the death rate from fatal rhabdomyolysis is about 0.15 death per million prescriptions [5,6]. The key features of rhabdomyolysis include acute renal injury secondary to deposition of myoglobin in the renal tubules resulting in case formation and compartment syndrome due to skeletal muscle injury and elevated biomarkers such as CK, myoglobin, deranged renal functions and elevated calcium and phosphate levels [5,6]. On the other hand, according to the US Food and Drug Administration Adverse Event Reporting System database, the incidence of statin-induced rhabdomyolysis is about 0.3-13.5 cases per 1,000,000 statin prescriptions [7].

The key management strategies in patients with rhabdomyolysis include include fluid resuscitation and urine alkalinization and occasionally, these patients with refractory hyperkalaemia, acidosis, fluid overload, or uremic complications require RRT [1].

## Case presentation

We present a case of a 57-year-old patient who presented to the hospital feeling unwell for the last couple of days. His past medical history (PMH) includes hypertension (HTN), diet-controlled type 2 diabetes mellitus (T2DM), previous renal failure requiring RRT in 1995 secondary to Ezetimibe, and hypercholesterolaemia. His regular medications include atenolol 50 mg once daily (OD), Felodipine 5 mg OD, and Atorvastatin 40 mg once at night (ON) for the last 20 years. He was triple vaccinated against COVID-19 and he had the last vaccine about 4-5 months ago. His clinical examination was unremarkable. His urine dip showed blood 3+, Protein 3+, leukocytes 3+, and nitrites negative. His lab tests showed creatinine 165 μmol/L, urea 8.5 mmol/L, white cell count 17.5 μL, Neutrophils 16.5 μL, C-reactive protein 39 mg/dL, CK level 28,289 U/L, lactate dehydrogenase (LDH) level >1,800 U/L as shown in Table 1. 

**Table 1:** Lab test results trend for patient

Blood test	Normal value	Day 1	Day 3	Day 6	Day 10	Day 18
White cell count	(4.0-11.0) 10^9^/L	17.5	17.2	12.5	11.2	7.5
Neutrophil	(1.7-7.5) 10^9^/L	16.5	16.0	11.5	10.2	6.4
Platelet	(150-400) 10^9^/L	186	172	232	235	250
Sodium	(133-146) mmol/L	136	138	137	135	139
Potassium	(3.5-5.3) mmol/L	4.5	4.6	4.5	4.3	4.0
Urea	(2.5-7.8) mmol/L	8.5	4.5	3.2	13.5	7.2
Creatinine	(61.9 to 110) µmol/L	165	350	699	265	85
Bicarbonate	22-29 mmol/l	17	16	17	18	22
Creatinine Kinase	40-320 U/L	28,289	135400	22500	3250	195
C reactive protein	<5 mg/L	39	89	95	55	25

His venous blood gases showed metabolic acidosis with Ph 7.32, PCO_2_ 4.55, PO_2_ 4.7, Bicarbonate (HCO_3_) 18.0, base excess (BE) -8.2, lactate 1.2, blood sugar (BM) 7.5 as shown in Table 2.

**Table 2:** Venous blood gases results

Blood test	Normal value	Day 1	Day 5	Day 8
Ph	7.35-7.45	7.32	7.33	7.31
Bicarbonate	22-29 mmol/L	18	17	16
Glucose	3.6-5.3 mmol/L	7.5	5.3	5.2
Lactate	0.5-2.2 mmol/L	1.2	1.3	1.4
PCO_2_	4.6-6.4 kPa	4.55	4.7	6.2
PO_2_	11.0-14.4 kPa	4.7	5.4	6.2
Base excess	2-3 mmol/L	-8.2	-9.2	-8.9

**Figure 1:**
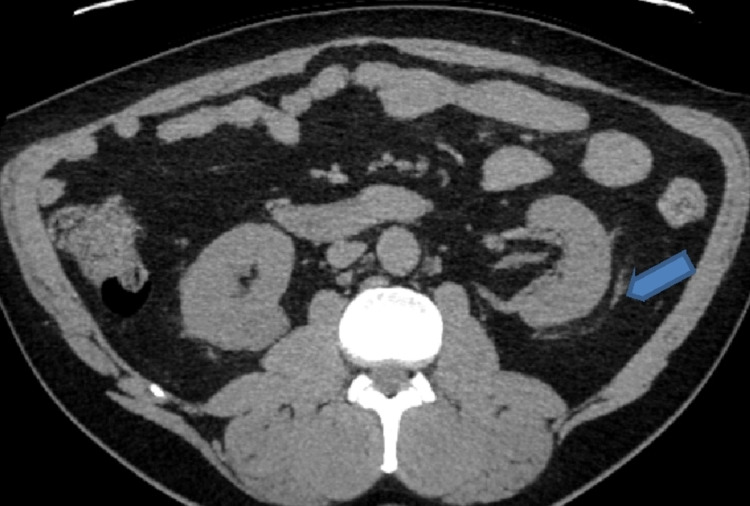
CTKUB Showing perinephric stranding in the left Kidney likely inflammatory or infectious in origin CTKUB - Computerized tomography scan of kidneys, ureters, and bladder

**Figure 2:**
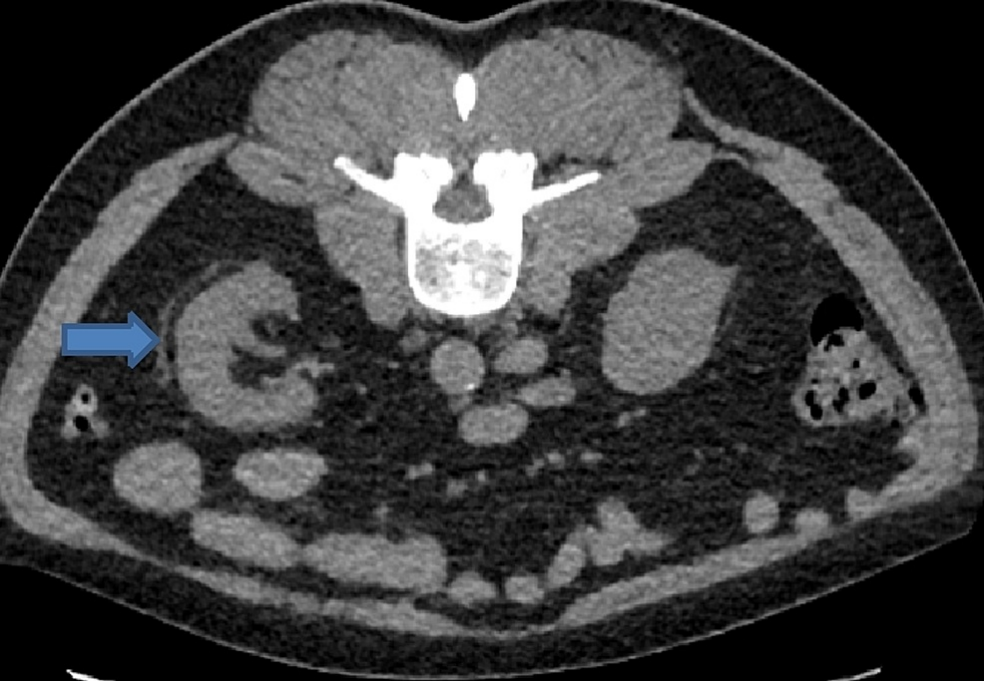
CTKUB showing perinephric stranding in the right kidney likely inflammatory or infectious in origin CTKUB - Computerized tomography scan of kidneys, ureters, and bladder

He had computerized tomographic scan of Kidneys, ureters and bladder (CTKUB) that showed bilateral renal perinephric stranding, particularly in the right kidney and a 6 mm non-obstructing calculus in the upper pole of left kidney as shown in Figures 1, 2.

His chest radiograph did not reveal any abnormality. He was apyrexial and his blood pressure (BP) was 113/65, heart rate was 73 beats per minute and oxygen saturation (SPO_2_) was 97% on room air (RA). He was commenced on intravenous fluids and was seen by nephrology team. He was also commenced on oral sodium bicarbonate 500 mg BD. His CK level continued to rise and climbed up to 135,400 on day 3 and started to come down on day 5 and came down to 11,500 on day 7. His catheter got blocked on day 5 and did start draining despite flushing and his catheter was changed. An urinary bladder scan showed only 650 mL urine in the bladder and is unlikely to have contributed to any worsening of renal function. The likely explanation for catheter blockage is tubular cast formation secondary to myoglobin deposition in renal tubules. His renal functions continued to deteriorate and creatinine went up from 165 μmol/L on admission to 895 μmol/L on day 7 and urea climbed up to 38mmol/L. His urine output gradually declined and he was passing under 25 mL urine an hour on day 6 despite having about 3-4 litres of fluids daily. He remained in positive fluid balance of about 1,500-2,000 mL all these days. He remained acidotic on blood gases and his bicarbonate level remained static at about 15-16. He was commenced on intravenous sodium bicarbonate 1.26% infusion one litre infusion over eight hours. He started to desaturate on day 6 of admission and his chest radiograph showed mild bilateral patchy infiltrates and his oxygen saturation was dropping to 84-85% on air. He was commenced on three litres of oxygen to maintain his saturation above 94%. His COVID-19 polymerase chain reaction (PCR) test was positive on day 6 although his initial COVID-19 swab which was also a PCR was negative and he had COVID-19 contact during his hospital admission as several patients tested positive on the ward. His repeat blood showed worsening renal function and his inflammatory markers were rising further. He was commenced on dexamethasone 6.6 mg IV OD and renal dose Co-Amoxiclav. He was given further sodium bicarbonate infusion and was discussed with nephrology team and patient underwent haemodialysis on day 9. His creatinine improved to 265 μmol/L and urea came down to 13.5 mmol/L on day 10. He required another session of renal replacement therapy on day 12 and his renal functions started to improve and returned to normal level on day 18 as shown in Table 2. He did not require any further RRT after the second session. He only received dexamethasone for COVID-19 infection and did not require any other antiviral therapy.

He had a similar episode back in 1995 when he developed renal failure secondary to Ezetimibe tablets. His viral hepatitis, autoimmune screen and vasculitis screen was all negative. This is perhaps the first case report where a patient developed renal failure secondary to lipid-lowering therapy twice in two decades. He was discharged home and had outpatient follow-up with nephrology team and his renal function has remained normal. He was also referred to specialist lipid clinic in view of renal failure secondary to lipid lowering therapy; unfortunately, we do not have information on the outcome of that. His atorvastatin was permanently stopped during this admission.

## Discussion

Rhabdomyolysis secondary to statins leading to renal failure requiring RRT is quite rare. A large United Kingdom (UK) based cohort study based on patients from general practice between 1991 and 1997 reported the mean incidence of statins induced myopathy to be 1.2 per 10,000 person-years [8]. Another large study reported this incidence in patients on monotherapy with atorvastatin, pravastatin, or simvastatin to be 0.44 and for cerivastatin was 5.34 [9]. It is important to mention that the clinical presentation of statins-induced myopathy can vary significantly and may present as myalgia, myositis, rhabdomyolysis, or asymptomatic increase in creatine kinase concentration [10]. 

The symptoms from statin-induced myopathy can vary from fatigue, muscle pain or tenderness, muscle weakness or cramps and the average duration for the onset of symptoms in patients newly started on statins was about six months in a small retrospective study, and symptoms resolved on average in about two months in patients after stopping statins [10]. The exact mechanism of statin-induced myopathy is unclear and there are three possible proposed mechanisms that could explain this. The first mechanism includes impaired cholesterol synthesis leading to in the myocyte membrane cholesterol structure and behaviour of membrane and the second mechanism is an impaired synthesis of compounds in the cholesterol pathway resulting in impaired enzyme activity in mitochondria. The third possible mechanism includes depletion of isoprenoids, a lipid-based product of the HMG-CoA reductase pathway preventing myofibre apoptosis.

The risk of myopathy is closely correlated with the dose of statins and is independent of reductions in low-density lipoprotein cholesterol [10]. The most common risk factors associated with myopathy include advanced age, female sex, low body mass index (BMI), impaired renal and hepatic function, polypharmacy or drug interactions or significant comorbidities, alcohol excess, infections, hypothyroidism, surgery or trauma, and dietary effects [10]. The symptoms of myopathy secondary to statins can disappear in about two months and this was also shown in the PRIMO study [11]. Most patients with myopathy do not require dialysis however a small proportion needs dialysis mainly if they are acidotic, have resistant hyperkalaemia, and/or do not respond to medical therapy [1]. The mainstay of management is medical or supportive therapy such as fluids and urinary alkalization and patient who have several renal impairments needs good hydration and alkalization of urine may be beneficial to minimize the risk of renal failure by using 1.26% or 1.4% sodium bicarbonate solution and occasionally mannitol.

Bicarbonate makes the urine alkaline thus reducing redox-cycling, lipid peroxidation, and myoglobin cast formation [11]. It is possible that urinary alkalization prevents renal failure and it can be used to prevent acute kidney injury (AKI) by increasing the urinary pH above 6.5 and can also be used to correct metabolic acidosis. It is important to be mindful of the potential problems that may arise with sodium bicarbonate such as paradoxical intracellular acidosis and volume overload in patients who are at higher risk of respiratory or circulatory collapse [11,12]. The other medical treatment used in patients with rhabdomyolysis induced renal failure is mannitol and it has been suggested that it improves renal perfusion, improved diuresis, increased renal perfusion, excretion of myoglobin, and a direct antioxidant effect on renal parenchyma [13]. Nevertheless, it’s important to be aware of the potential side effects of mannitol such as pre-renal azotemia and volume depletion, and should only be in patients in whom fluid intake is not enough to get 300 mL/hour urine daily and should be avoided in anuric patients [12,13].

There are several studies published on statin-induced rhabdomyolysis and renal failure requiring RRT [3,5,7]. The overall incidence of the disease is small and one study suggested the number needed to treat to observe one case of rhabdomyolysis on statin alone is 22,727 and 484 in diabetic patients who were on combined therapy of statin and fibrates. The number needed to treat (NNT) to observe one case of rhabdomyolysis was 22,727 for statin monotherapy and 484 for older patients with diabetes mellitus taking both a statin and a fibrate.

Statin-induced rhabdomyolysis has also been reported in patients with hypothyroidism and one such case report is based on the presentation of a 74-year-old patient who presented with swelling in his lower limbs, stiffness in his muscles and responded to intravenous fluid hydration and urinary alkalization [1]. Another case report is based on the presentation of 72 years old Sri Lankan patient with a background of high cholesterol, type 2 diabetes mellitus, chronic kidney disease, and hypothyroidism. He presented to Emergency Department with rhabdomyolysis secondary to combination therapy of statin and gemfibrozil [2]. Another 67-year-old man who was on atorvastatin after suffering myocardial infarction (MI) four months ago presented with statin-induced hepatitis, rhabdomyolysis, and AKI and unfortunately passed away [13,14].

In patients with severe rhabdomyolysis, conventional haemodialysis is usually not enough due to its failure to remove the bigger size myoglobin particles, and patients with severe acidosis, fluid overload or refractory hyperkalaemia, require intermittent RRT and renal functions usually return to normal after few sessions of RRT without requiring further RRT [3,12].

## Conclusions

In conclusion, statin-induced rhabdomyolysis is a rare serious adverse effect of statins resulting in renal failure and patients may require RRT. The risk is increased with combination lipid-lowering therapy and these patients may present with severe rhabdomyolysis which results in renal failure. Our patient case is unique due to the fact that he had severe rhabdomyolysis twice in two decades despite being on two different lipid-lowering therapy and required intermittent haemodialysis both times. Our patient recovered well from the episode and his renal functions returned back to a normal level.
